# Internalization of Appearance Ideals and Not Religiosity Indirectly Impacts the Relationship Between Acculturation and Disordered Eating Risk in South and Southeast Asian Women Living in the United States

**DOI:** 10.3389/fpsyg.2022.843717

**Published:** 2022-07-18

**Authors:** Sonakshi Negi, Erik M. Benau, Megan Strowger, Anne Claire Grammer, C. Alix Timko

**Affiliations:** ^1^Department of Child and Adolescent Psychiatry and Behavioral Sciences, Children’s Hospital of Philadelphia, Philadelphia, PA, United States; ^2^Department of Psychology, University of Kansas, Lawrence, KS, United States; ^3^Department of Psychology, SUNY Old Westbury, Old Westbury, NY, United States; ^4^Department of Behavioral and Social Sciences, University of the Sciences, Philadelphia, PA, United States; ^5^Department of Psychology, Old Dominion University, Norfolk, VA, United States; ^6^Department of Psychiatry, Washington University School of Medicine, St. Louis, MO, United States; ^7^Department of Psychiatry, Perelman School of Medicine, University of Pennsylvania, Philadelphia, PA, United States

**Keywords:** body dissatisfaction, thin ideal, Asian identity, religiosity, path analysis

## Abstract

**Objective:**

Studies that examine disordered eating in samples of Asian individuals living in the United States frequently combine all individuals of Asian descent into a single group, which can obscure important differences between groups and their experiences of acculturation. The goal of the present study was to establish the relation of acculturation, internalization of appearance ideals, and religiosity as predicting body dissatisfaction and disordered eating in women of South and Southeast Asian (SSEA) descent.

**Method:**

Women of SSEA descent (*N* = 112) aged 18–51 years (*M* = 23.10, *SD* = 6.4) completed a battery of questionnaires that inquire about these variables. A path analysis was conducted with acculturation serving as the independent (exogenous) variable, religiosity and internalization of the thin ideal as mediators, and body dissatisfaction and disordered eating as dependent (endogenous) variables.

**Results:**

Direct paths from acculturation to both body dissatisfaction and disordered eating were not significant. Thin ideal internalization completely accounted for the path from acculturation to both endogenous variables; whereas, religiosity did not significantly account for any indirect effect.

**Discussion:**

For SSEA women, internalization of appearance ideals is a potentially greater risk factor for disordered eating than acculturation or religiosity. As this was an atemporal mediation analysis, more work needs to be done exploring predictors of internalization in this population and how that may impact the development of disordered eating.

## Introduction

There has been an increased call to diversify research and scholarship in the field of eating disorders (Burke et al., 2020; Huryk et al., 2021). Despite these calls, the majority of research on disordered eating has been conducted in samples of predominantly affluent White girls and women (Rodgers et al., 2018; Huryk et al., 2021), contributing to a misconception that disordered eating is a “rich, White, and female problem” (Brown et al., 2009). When racial and ethnic diversity are considered in this research, they are often analyzed as “White” vs. “People of Color/Ethnic Minorities” (e.g., Rodgers et al., 2017) or as “immigrant” vs. “native-born” member of a culture (Doris et al., 2015), putatively for ease of presentation or due to underpowered samples that prevent more fine-grained analyses. Despite being the fastest growing demographic in the United States (Budiman and Ruiz, 2021), individuals of Asian descent are infrequently considered in disordered eating research, and, when included, are often analyzed as a unified group (“Asian”). A singular “Asian” category ignores or obfuscates potentially informative sociocultural and psychosocial variation across Asian cultures, which limits our understanding of eating pathology in these populations (Goel et al., 2021).

In the United States, individuals of South and Southeast Asia (SSEA)^1^ and East Asian^2^ descent are the groups most frequently designated as “Asian,” as Central Asia and Western Asia are often designated as “Other,” White, or Middle Eastern.^3^ Governmental guidance is often cited as justification for aggregating individuals of Asian descent into a single category (e.g., Office of Management and Budget, 1997). This guidance has long been criticized as outdated, ineffective, and otherwise diminishing the individuality of people from these countries (Panapasa et al., 2011; Holland and Palaniappan, 2012; Lee and Ramakrishnan, 2019; Hayes-Bautista et al., 2021). The conflation of East Asia and SSEA disregards markedly distinct histories and values that may impact eating disorder attitudes and behaviors (Komisarof and Leong, 2016). For SSEA, colonization infused many Western ideals into the culture, economy, politics, and media; whereas these aspects of East Asia were not nearly as impacted by colonization (Kwon, 2011; Leong-Salobir, 2011; Hasan, 2012). Not surprisingly, body and appearance ideals in SSEA cultures may be heavily influenced by European standards; whereas “Westernization” is not as prominent in East Asian culture (Getz, 2014; Swami, 2015). Religion and its role in culture also differentiates these regions. The predominant religions in SSEA cultures are Hinduism, Islam, and Christianity (Evers, 2015) in contrast to East Asia, which features predominantly Buddhist, Confucian, Daoist, and Shinto communities (Walker, 2012). Religious adherence to Christianity, Islam, and/or Hinduism has been identified as possible risks for disordered eating across cultures (Spangler, 2010; Akrawi et al., 2015; Gerber et al., 2015). In contrast, the association between religion and disordered eating in East Asia is minimal (Cummins et al., 2005; Getz, 2014).

### Impact of Acculturation

Cultural history and practices continue to shape eating behavior when individuals relocate to a new culture (Reddy and Crowther, 2007; Doris et al., 2015; Swami, 2015, 2016; Warren and Akoury, 2020). Women of SSEA descent living in predominantly White cultures report greater prevalence of eating disorder symptoms than their White counterparts (Cummins et al., 2005; Doris et al., 2015; Monterubio et al., 2020). Although prevalence rates are important, being of SSEA descent is not a risk factor in-and-of itself. Women of SSEA descent may have unique pathways to disordered eating risk that are relatively unexplored (Goel et al., 2021). Studies with ethnic minority populations provide evidence that acculturation—the adoption of “the attitudes, values, customs, beliefs, and behaviors” of the dominant culture (Abraido-Lanza et al., 2006, p. 1342)—is a key component to disordered eating onset (Doris et al., 2015; Rodgers et al., 2018). It is less clear whether, and how, acculturation relates to disordered eating for individuals of Asian descent, including those of SSEA descent. Previous work has found a direct correspondence of acculturation to disordered eating for East Asian women (Sussman et al., 2007; Huang, 2019). However, only *indirect* relations between acculturation and disordered eating have been identified for SSEA women (Iyer and Haslam, 2003; Reddy and Crowther, 2007; Franzen and Smith, 2009; Akoury et al., 2019).

Acculturation may be linked to body image dissatisfaction, or the negative self-evaluation of one’s body shape, weight, or physical appearance (Smolak, 2006). This relationship may be explained in part by internalization of appearance (typically thin) ideals, or the degree to which one accepts these ideal and intends to pursue them (Warren et al., 2013). Although the prevalence and intensity of disordered eating risk may not differ between White, East Asian, and SSEA women (Tareen et al., 2005), the form and function of these factors may differ between groups. For example, SSEA women report a fixation on, and desire for, lighter skin tone and hair color (Goel et al., 2021); whereas White women tend to focus on facial features, darker (tanned) skin, and reduced adiposity overall (e.g., Warren, 2012). Additionally, the fear of weight gain that can be part of the clinical diagnosis for anorexia nervosa is nearly absent in samples of East and SSEA women, though a desire to be thin is often prominent regardless of culture of origin (Tareen et al., 2005; Lee and Lock, 2007). Distinct to SSEA cultures is that food restriction is a sanctioned treatment for a variety of ailments, and thinness for health purposes is often cited as a motive for disordered eating and self-starvation (Nouri et al., 2011; Vaidyanathan et al., 2019; Goel et al., 2021). Importantly, acculturation may modulate and reverse these motives: for women of SSEA descent, greater acculturation is associated with internalization of and desire for, an ideal “Western” appearance for the same aesthetic motives as White women (Swami, 2015; Akoury et al., 2019; Goel et al., 2021).

### Impact of Religiosity

Recent work has identified religiosity—the degree to which one is motivated to practice religion due to internal and/or external motivation (Ladd and Spilka, 2013)— as a potential contributing factor for eating disorder risk (Akrawi et al., 2015). The majority of research on religiosity and disordered eating has focused on White, Judeo-Christian samples (Boyatzis and Quinlan, 2008; Richards et al., 2013) with comparatively little research into the same risk within Hinduism and Islam, religions that are predominant in SSEA populations (Allerton, 2009). Christian (mainly Protestant) adherence has been identified as a risk for eating disorders, though the mechanisms are not fully understood (Akrawi et al., 2015). Some suggest Christian adherence may frame food restriction as a means to avoid the “sin of gluttony,” control bodily urges, and/or to atone for sins (Spangler, 2010; Gerber et al., 2015). Limited available work within the Muslim population suggests that greater religious adherence is associated with greater eating disorder symptoms (e.g., Gulamhussein and Eaton, 2015; Thomas et al., 2018). Some suggest that dietary guidelines and religious fasting may trigger, disguise, or exacerbate disordered eating behavior (Cummins et al., 2005; Akgul et al., 2014; Pengpid et al., 2015; Vaidyanathan et al., 2019; Hasan et al., 2021). Other aspects of Hinduism and Islam may modulate risk for disordered eating. For example, modest dress for women as part of religious observance [including niqab (a face veil and head covering) or hijab (head covering) in Islam] may reduce or compensate for concerns about appearance, potentially due to reduced perceived objectification of others (Swami et al., 2014; Wilhelm et al., 2018). Given the important influence of religion in daily life and culture within the SSEA region (Allerton, 2009; Siebenhütter, 2019), it is critical to determine whether, and how, religiosity is associated with risk for disordered eating in this population.

### The Present Study

The main aim of the present study was to establish whether and how acculturation, religiosity, and internalization of appearance ideals are atemporally associated with body dissatisfaction and disordered eating in a sample of SSEA women living in the United States (Winer et al., 2016). We hypothesized that acculturation, religiosity, and internalization of appearance ideals would all have direct, positive associations with both body dissatisfaction and disordered eating. We further hypothesized that the indirect associations of internalization and religiosity would better account for the variance of the association of acculturation to body dissatisfaction and disordered eating, respectively. The goal was to establish the importance of internalization and religiosity to the relationship between acculturation and body dissatisfaction and disordered eating in SSEA women and provide a foundation upon which models could be built.

## Materials and Methods

### Participants

A total of 340 individuals completed consent and began participation. Participants were undergraduates recruited from two universities who received course credit for participation and community participants from the surrounding area responded to fliers and announcements online. Community participants were not compensated for participation. Participants were included if they were female, at least 18 years old, and of SSEA descent (based on self-identification, place of birth, and/or at least one parent’s place of birth). Respondents were removed from analyses if they: reported their sex as male (*n* = 19), did not report their sex (*n* = 2), were under 18 years of age (*n* = 10), were not of SSEA descent (*n* = 152).^4^ A final 45 participants were omitted from analyses for failing to complete the Suinn-Lew Asian Self-Identify Acculturation Scale (SL-ASIA) and at least one other questionnaire. The final sample consisted of 112 participants [97 (87%) South Asian, 14 (13%) Southeast Asian, 1 (<1%) both]. Given similar sociocultural backgrounds of South and Southeast Asian nations (described above), and that the two groups did not significantly differ on their Body Mass Index (BMI) or any of the measures of interest described below (*p*s > 0.4), we combined these two groups while acknowledging the limitations of doing so. Most (61%) of the sample was born in the United States, and all participants lived in the United States at the time of participation. An *a priori* power analysis using tools by Preacher and Coffman (2006) indicated that the Path Analysis (described below), could be minimally powered (1–β = 0.80) with 92 respondents. The present sample more than adequately powered the model (1–β = 0.91).

### Measures

#### Demographic Information

We collected age, race, sex, country of birth, parental country of birth, religion, and current weight (in pounds) and height (in inches) to calculate BMI [(Weight/Height^2^)× 703]. BMI was categorized based on standardized guidelines amended for Asian and South Asian individuals (Weir and Jan, 2021). Sex was collected as a forced-choice, binary item. Race was selected from a drop-down list with “Other” as a fill-in item. Remaining demographic items were entered as fill-in text. See Table 1.

**TABLE 1:** Demographic information.

Participant country of birth	*N* (%)	
Bangladesh	4 (4)	
India	19 (17)	
Pakistan	11 (10)	
Philippines	4 (4)	
Singapore	1 (1)	
United Kingdom	2 (2)	
United States	68 (61)	
Other^a^	3 (3)	
**Parental birth country**	**Mother**	**Father**
Bangladesh	6 (5)	5 (5)
Burma	0 (0)	1 (1)
Cambodia	1 (1)	1 (1)
India	63 (56)	61 (55)
Laos	3 (3)	1 (1)
Pakistan	19 (17)	22 (20)
Philippines	4 (4)	6 (5)
Thailand	0 (0)	2 (2)
United States	1 (1)	0 (0)
Vietnam	7 (6)	7 (6)
Other	3 (3)^b^	2 (2)^c^
No Response	4 (4)	4 (4)
**Religion**		
Muslim	45 (40)	
*N* (%) of Muslims who wear Hijab	19 (44)	
*N* (%) of Muslims who wear Niqab	1 (2)	
Hindu	28 (25)	
Christian^d^	20 (18)	
Buddhist	6 (5)	
Jewish	1 (1)	
Atheist	4 (4)	
Other^e^	7 (7)	
**BMI**	**M (SD)**	
Western Born (United States or United Kingdom)	22.17 (3.74)	
Born elsewhere	24.36 (5.12)	
Sample	22.99 (4.42)	

*All values are N (%) except as noted.*

*^a^Japan (1), Kenya (1), and Tanzania (1).*

*^b^“Africa” (1), Zambia (1), Tanzania (1).*

*^c^“Africa” (1), Tanzania (1).*

*^d^Includes five who specified “Catholic” or “Roman Catholic”.*

*^e^Sikh (2), Jain (4), Hindu and Jain (1).*

#### Disordered Eating Symptoms

The 26-item Eating Attitudes Test (EAT-26; Garner et al., 1982) is a self-report measure of disordered eating and risk for eating pathology in clinical and non-clinical samples. Items are scored on a Likert-type scale ranging from 1 (*Never*) to 6 (*Always*). Items include “I find myself preoccupied with food” and “I like my stomach to be empty.” Greater scores indicate greater preoccupation with eating and weight loss and, thus, disordered eating risk. This instrument has been used in previous samples of SSEA women living in the United States (e.g., Iyer and Haslam, 2003; Reddy and Crowther, 2007). We used the sum-score of the EAT-26, which has previously shown validity and reliability in non-clinical samples (e.g., Rogoza et al., 2016). The present sample exhibited excellent internal consistency on the sum score of this measure (α = 0.91).

#### Body Image Dissatisfaction

The Body Shape Questionnaire (BSQ; Cooper et al., 1987) is a 34-item self-report instrument that assesses multidimensional aspects of body shape dissatisfaction. Items are scored 1 (*never*) to 6 (*always*). Items include “Have you felt ashamed of your body?” and “Has worry about your shape made you diet?” Greater scores indicate greater dissatisfaction with one’s body. The BSQ has been used frequently with samples of SSEA respondents (Vaidyanathan et al., 2019), though the questionnaire’s psychometrics have not yet been systematically verified for cross-cultural use (Kling et al., 2019). Internal consistency for this measure was excellent (α = 0.98).

#### Internalization of Appearance Ideals

We used the Sociocultural Attitudes Toward Appearance Questionnaire-Third Edition^5^ (SATAQ-IG; Thompson et al., 2004) to measure internalized pressures for an ideal appearance. For the purposes of the present study, we included only the nine-item “Internalization-General” subscale as our hypotheses center on internalization of an ideal appearance from media and the dominant culture. Items on the scale include “I would like my body to look like the models who appear in magazines” and “I’ve felt pressure from TV or magazines to change my appearance.” Greater scores suggest greater internalization of appearance ideals. Notably, though this subscale *implies* a thin appearance, the questions that comprise this measure discuss “appearance” broadly, including some questions about the body. Therefore, this scale does not discuss a drive for thinness, specifically. Previous work supports the validity and reliability of SATAQ scores in samples of Asian women from multiple regions (e.g., Warren et al., 2013; Swami et al., 2014). Items are scored on a five-point Likert-type scale ranging from 1 (*completely disagree*) to 5 (*completely agree*). The SATAQ-IG had good internal consistency (α = 0.83).

#### Acculturation

We measured acculturation using the 26-item Suinn-Lew Asian Self-Identify Acculturation Scale (SL-ASIA; Suinn et al., 1987, 1992). SL-ASIA self-report scale that assesses how assimilated an individual is into Western culture in a variety of areas. The SL-ASIA has previously demonstrated validity and reliability in measuring acculturation within samples of individuals from diverse Asian countries (Kodama and Canetto, 1995; Phillips et al., 2016). Items are scored on a scale of 1 (low acculturation) to 5 (high acculturation). For example, the question “How would you rate yourself” contains the options of 1 “very Asian” to 5 “very Westernized”; the question “What is your music preference” contains the options of 1 “Only Asian music…” to 5 “English only.” Higher scores indicate greater self-identified acculturation. Items are written to be applicable across Asian populations in the United States and has shown adequate psychometric properties in previous samples of South Asian respondents (Iyer and Haslam, 2003). Internal consistency was acceptable for the total score (α = 0.77).

#### Religiosity

The Feagin Intrinsic/Extrinsic Religiosity Scale (FIERS; Feagin, 1964) is a 21-item self-report questionnaire that measures religiosity based on intrinsic (e.g., personally meaningful) and extrinsic (instrumental or engaged due to external pressure) motives. The first 18 items are scored on a scale of 1 (*strongly disagree*) to 5 (*strongly agree*), one item (“I read literature about my religion”) scored on a scale of 1 (*rarely*) to 4 (*at least once a week*), one (“If not prevented by unavoidable circumstances, I attend religious services”) scored as 1 (*never*) to 7 (*every day*), and one (“If I were to join a group affiliated with my religious community, I would prefer to join:”) was scored as 1 (*a social fellowship or club*) or 2 (*an educational study group*). Thus, possible scores range from 18 to 103, where higher scores correspond to greater religiosity. We used the sum score of the instrument to attain a measure of overall measure of motives for engaging in religious observance. Like previous work with samples of diverse religions (e.g., Hill and Dwiwardani, 2010) or Asian participants (e.g., Fischer et al., 2016), we modified items to be inclusive (e.g., “attending religious services” replaced “attending Church”). Thirteen participants did not respond to one item from this inventory. Little’s test suggested that the items were missing completely at random (MCAR), meaning there was no consistent pattern of omitted responses based on the other variables included in this study, χ^2^ (166) = 163.56, *p* = 0.539. We replaced missing values using person-mean imputation wherein the mean of a respondent’s remaining items (rounded to an integer) replaced the missing datum (Acock, 2005). Optimal data replacement methods are not established, and person-mean imputation has been found to be acceptable when data are missing non-systematically (i.e., MCAR, as in the present set) the number of missing items is small (in this case, ≈5% per participant) and the number of participants needing replaced data are infrequent (in this case, ≈12% of participants) (Downey and King, 1998; Huisman, 2000; Eekhout et al., 2014). Note that the FIERS was the only measure with missing items in the current dataset and it is unclear why. After item replacement, internal consistency on the scale was good (α = 0.90). We also asked if participants wore a hijab, niqab, burqa [fully enveloping outer garment], or other form of modest dress and their reasons for doing so.

### Procedure

After completing informed consent, participants completed a series of questionnaires on their own devices, presented in a standardized order *via* an online website (SurveyMonkey Audience, Survey Monkey, Inc., San Mateo, CA, United States). This study was approved by all appropriate ethics review committees.

### Data Analysis

#### Missing Data

Three respondents reported implausible height and weight to calculate BMI: two respondents reported their weight as <60 pounds (27 kg) and one reported their height as 10 inches (0.25 m). When conducting the path analyses, rather than delete these cases (to retain power and proceed with bootstrapping^6^), we replaced the three implausible BMI values with the sample mean. For all other analyses, the implausible BMI values were omitted. Compared to those who provided complete and plausible BMI scores, the individuals who provided implausible BMI values did not significantly differ on any available measure or demographic variable (*p*s > 0.052). The FIERS was the only instrument that was missing specific items (the imputation of which is discussed above). A separate 15 respondents omitted one *entire* questionnaire: 13 FIERS, one BSQ, and one SATAQ-IG. No included respondent missed more than one questionnaire. The 13 individuals who did not complete the FIERS at all were separate from the 13 whose data were imputed. Little’s test was significant, χ^2^ (16) = 35.75, *p* = 0.003, suggesting that questionnaire data were not MCAR. Follow-up tests indicated that completers of the FIERS (*n* = 99) were significantly younger (*M* = 22.28, *SD* = 5.46) than non-completers (*n* = 13; *M* = 29.31, *SD* = 8.63), *t*(110) = 4.04, *p* < 0.001, *d* = 1.19. Data from available completers indicate that age was not meaningfully related FIERS scores (*r* = 0.033; see Table 2). Results of the remaining *post-hoc t*-tests and χ^2^ tests were not significant, *p*s > 0.3. As a result, these data can be diagnosed as missing at random (Acock, 2005; Mack et al., 2018). We did not examine non-completers of the SATAQ-IG or BSQ given there was one participant each. Individuals with missing data were therefore retained in an all analyses as appropriate (discussed below).

**TABLE 2:** Descriptive statistics and Pearson correlations for each variable of interest.

		Complete responses	M (SD)	1	2	3	4	5	6	7	8
1	SL-ASIA	112	62.13 (8.44)	–							
2	SATAQ-IG	111	28.06 (8.13)	0.245**	–						
3	FIERS	99^a^	57.62 (12.67)	0.090	–0.066	–					
4	BSQ	111	92.40 (42.30)	0.168	0.569***	0.091	–				
5	EAT	112	12.16 (11.61)	0.104	0.423***	–0.026	0.702***	–			
6	BMI	109^b^	22.99 (4.48)	–0.008	–0.063	–0.072	0.248**	0.056	–		
7	Age	112	23.10 (6.28)	–0.316**	–0.231*	0.033	–0.148	–0.132	0.328**	–	
8	Born West. [*N*, (%)]	112	70 (62.50)	–0.443***	–0.027	–0.043	–0.148	–0.048	0.245**	0.460***	–

*SL-ASIA, Suinn-Lew Asian Self Identity Acculturation; SATAQ-IG, Sociocultural Attitudes Toward Appearance Questionnaire-Internalization General subscale; FIERS, Feagin Intrinsic-Extrinsic Religiosity Scale; BSQ, Body Shape Questionnaire; EAT, Eating Attitudes Test; BMI, Body Mass Index; Born West., whether participant was born in the United States (n = 68) or United Kingdom (n = 2) coded as 1 = Yes, 2 = No (negative coefficients indicate greater scores for “Western”-born individuals).*

*^a^Includes 13 surveys with person-mean imputed data (separate from the 13 missing FIERS responses).*

*^b^Three values were omitted from these analyses and this table for being implausible.*

*p < 0.05, **p < 0.01, ***p < 0.001.

#### Correlations and Path Analyses

**FIGURE 1:**
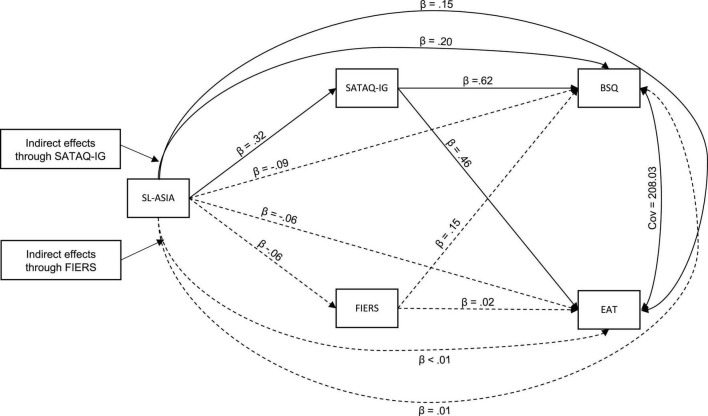
Model used in the SEM analyses. Straight lines indicate direct paths; curved single-arrow lines indicate indirect paths; solid lines indicate a significant path (*p* < 0.05); dashed lines indicate non-significant paths (*p* > 0.05) based on unstandardized coefficients. Standardized coefficients for direct effects (β) are presented. SL-ASIA, Suinn-Lew Asian Self-Identify Acculturation Scale; SATAQ-IG, Sociocultural Attitudes Toward Appearance Questionnaire-Internalization General subscale; FIERS, Feagin Intrinsic-Extrinsic Religiosity Scale; BSQ, Body Shape Questionnaire; EAT, Eating Attitudes Test. Note that paths for BMI and US-Born are not presented.

First, we conducted Pearson correlations for all variables of interest (Table 2). We also conducted *t*-tests within the sample of Muslim women to determine if there were significant differences in the variables of interest as a function of wearing a hijab (*n* = 19) or niqab (*n* = 1); the 20 hijab or niqab wearers were combined into a single group. To address the main hypotheses of this study, we conducted a path analysis wherein we entered the SL-ASIA as the exogenous (independent) variable, scores of the EAT and BSQ as the endogenous (dependent) variables. The FIERS and SATAQ-IG were entered as mediators. We drew direct paths from the SL-ASIA to the FIERS and SATAQ-IG, and from the FIERS and SATAQ-IG to the EAT and BSQ (see Figure 1). We conducted a path analysis rather than multiple regression models to reduce the likelihood of Type I error and to control for variance associated with each path in the model (Iacobucci et al., 2007; Zhao et al., 2010). Cut-offs for fit-indices of the model were based on the well-established scores presented by Hu and Bentler (1999).^7^ Given the model contains just one degree of freedom, these indices are likely not especially meaningful, and are presented here to verify the fit of the residuals to these paths. Based on the recommendations of Shrout and Bolger (2002), effect size (based on standardized estimates) can be understood as small (direct β > 0.10; indirect β > 0.01), medium (direct β > 0.30; indirect β > 0.09), or large (direct β > 0.50; indirect β > 0.25).

We allowed the residuals of the BSQ and EAT to correlate as they were highly parallel (Acock, 2013; see Table 2). Since data were missing at random, we used maximum likelihood with missing values, which retains all participants and calculates path coefficients without imputation or further adjustments (Acock, 2013). Confidence intervals and statistical significance were calculated with bias corrected bootstraps using 1000 replications. After assessing the direct effects, we tested the bootstrapped indirect effect based on guidelines suggested by Zhao et al. (2010), an extension of those of Preacher and Hayes (2004, 2008). The Zhao et al. (2010) procedure compares the bootstrapped indirect effect to the direct effect (in this case, using 5000 Monte Carlo replications). If the direct effect is not significant but the indirect effect is significant, this is considered “indirect-only” (or “full”) mediation, if both the indirect and direct effects are significant, then this is considered “partial” mediation (either “complementary” or “competitive” mediation, depending on the direction of the effect). Of course, if neither the direct nor indirect effects are significant, this is “no-effect non-mediation,” and if the direct effect is significant but the indirect effect is not, this is “direct effect non-mediation.”

Previous work suggests that BMI and country of birth are associated with reported body shape dissatisfaction and disordered eating, including in East Asian and SSEA populations (Cummins et al., 2005; Gutin, 2018). BMI and country of birth also correlated with some of the variables of interest (Table 2). To control for these possible confounds, we entered BMI and birthplace of respondent (dummy coded as “Western” vs. other^8^) as exogenous variables with the same paths as the SL-ASIA. For ease of presentation, we omitted the paths of country of birth and BMI from Figure 1 and the values from Table 3, though these are presented in Supplementary Appendices A, B. We used Stata 17 (Stata, Inc., College Station, TX, United States) and the *medsem* package for STATA version 1.0 (Mehmetoglu, 2018).

**TABLE 3:** Summary statistics for direct and indirect effects in the path analysis.

	B	SE of B	β	*z*	95% CI Lower	95% CI Upper
**Direct effects**						
SL-ASIA→						
SATAQ-IG	0.31***	0.08	0.32	3.83	0.15	0.48
FIERS	0.15	0.19	0.10	0.76	–0.23	0.53
BSQ	–0.46	0.51	–0.09	–0.9	–1.46	0.54
EAT	–0.08	0.17	–0.06	–0.45	–0.42	0.26
SATAQ-IG→						
BSQ	3.28***	0.40	0.62	8.15	2.49	4.07
EAT	0.65***	0.15	0.46	4.33	0.36	0.95
FIERS→						
BSQ	0.51	0.30	0.15	1.69	–0.08	1.10
EAT	0.02	0.08	0.02	0.24	–0.13	0.17
**Indirect effects**						
SL-ASIA→						
SATAQ → BSQ	1.03***	0.29	0.20	3.62	0.47	1.59
SATAQ → EAT	0.21**	0.07	0.15	2.89	0.07	0.35
FIERS → BSQ	0.07	0.11	0.01	0.69	–0.14	0.28
FIERS → EAT	<0.01	0.01	<0.01	0.22	–0.02	0.03
**Covariance**						
EAT → BSQ	208.03***	38.85	–	5.35	131.89	284.17

*95% CI and statistical significance were calculated with bias corrected bootstraps using 1000 replications on the unstandardized values.*

*B, unstandardized coefficient; β, standardized coefficient; SL-ASIA, Suinn-Lew Asian Self-Identity Acculturation; SATAQ-IG, Sociocultural Attitudes Toward Appearance Questionnaire-Internalization General subscale; FIERS, Feagin Intrinsic-Extrinsic Religiosity Scale; BSQ, Body Shape Questionnaire; EAT, Eating Attitudes Test.*

**p < 0.01, *** p < 0.001.

## Results

### Sample Characteristics and Questionnaire Correlations

Demographic information for the sample is presented in Table 1; means, standard deviations, and correlations for all instruments can be found in Table 2. As shown in Table 1, most (61%; *n* = 68) of the sample was born in the United States followed by India (17%; *n* = 19) and Pakistan (10%; *n* = 11). The remaining sample was born across a diverse set of countries. The median age of the sample 21.0 years (*M* = 23.10, *SD* = 6.28, range: 18–51); 90% were <30 years old. Of those who provided a plausible height and weight (*n* = 109), BMIs ranged from 16.14 to 40.74 kg/m^2^ (*M* = 22.99, *SD* = 4.48). About half of the sample (*n* = 54; 50%) was in the “normal” BMI range (18.5–25 kg/m^2^), 12 (11%) were “underweight” (BMI < 18.5 kg/m^2^), and 43 (40%) were “overweight” or “obese” (BMI > 23 kg/m^2^). Results of Pearson correlations showed that the SL-ASIA scores positively correlated with the SATAQ-IG. The SATAQ-IG, in turn, positively correlated with the EAT and BSQ. The FIERS did not significantly correlate with any variable. Of the Muslim women in the sample (*n* = 45), those who wore a Hijab or Niqab (*n* = 20) did not significantly differ from those who did not (*n* = 25) on BMI or questionnaire data, including the FIERS (*p*s > 0.22, *d*s < 0.38), and were statistically matched for country of birth (Western vs. elsewhere), χ^2^ (1) = 3.04, *p* = 0.082, *V* = 0.260 (see Supplementary Appendix C). The most common religious identity was Muslim (*n* = 45; 40%), followed by Hindu (*n* = 28; 25%), and Christian (*n* = 20; 18%). There were some significant main effects of religious identity on the variables of interest (on the FIERS, age, and birthplace) that are presented and discussed in Supplementary Appendix D.

### Path Analysis

#### Test of Model Fit

The model achieved a near perfect fit for the data: χ^2^ (1) = 1.01, *p* = 0.316, RMSEA = 0.01 [90% CI_RMSEA_ (<0.01, 0.25)], *pclose* = 0.380, CFI = 1.00, TLI = 1.00. The model fit about 32% of the data overall (*R*^2^ = 0.318), about 50% of the variance for the BSQ (*R*^2^ = 0.490), and about 21% of the variance for the EAT (*R*^2^ = 0.210).

#### Direct and Indirect Effects

Figure 1 presents the results of the path analysis. Solid lines indicate a significant path (*p* < 0.05) whereas dashed lines indicate a path that was not significant. Table 3 presents the direct and indirect statistics for variables of interest in the model.

#### Body Shape Concerns

The direct path from the acculturation (SL-ASIA) to the body shape dissatisfaction (BSQ) was not significant. However, the paths from the SL-ASIA to internalization (SATAQ-IG) and from the SATAQ-IG to the BSQ were significant and each path corresponded to large effect sizes. Finally, the indirect path from the SL-ASIA to the BSQ with the SATAQ-IG as the mediator was significant and corresponded to a medium effect size *B* = 1.03 (β = 0.20), *SE* = 0.30, *z* = 3.42, *p* = 0.001. Since the direct effect was not significant, *B* = –0.46 (β = 0.09), *p* = 0.370, there was indirect-only (full) mediation. The indirect effect of the SATAQ-IG (1.03) was about 179% as large as the total effect (0.576), and 2.3 times as large as the direct effect of SL-ASIA to BSQ (0.458).

Unlike with the SATAQ-IG as the mediator, the paths from the SL-ASIA to religiosity (FIERS), and from FIERS to the BSQ, were not significant. The indirect (Monte Carlo) path from the SL-ASIA to the BSQ with the FIERS as a mediator was not significant, *B* = 0.07, *SE* = 0.12 (β = 0.01), *z* = 0.59, *p* = 0.558, nor was the direct effect, *B* = –0.46 (β = 0.09), *p* = 0.370. Therefore, there was “no effect, non-mediation.” The indirect effect of the FIERS (0.074) was about 19% of the total effect (0.383), and 0.2 times as large as the direct effect of SL-ASIA to BSQ (0.458).

#### Disordered Eating

Like the BSQ, the direct path between the SL-ASIA and eating disorder symptoms (EAT), was not significant. The paths from the SL-ASIA to the SATAQ-IG, from the SATAQ-IG to the EAT were significant. As with the BSQ, the indirect (Monte Carlo) test was significant, and corresponded to a medium effect size, *B* = 0.21 (β = 0.15), *SE* = 0.07, *z* = 2.84, *p* = 0.005. Since the direct effect was not significant, *B* = –0.08 (β = –0.06), *p* = 0.650, the results indicate an indirect-only (full) mediation. The indirect effect of the SATAQ-IG (0.206) was about 161% as large as the total effect (0.128), and 2.6 times as large as the direct effect of SL-ASIA to EAT (0.078).

Again, like the BSQ, the direct paths from the SL-ASIA to FIERS and the EAT, and from the FIERS to the EAT were not significant. The indirect (Monte Carlo) test was not significant, *B* < 0.01 (β = 0.06), *SE* = 0.02, *z* = 0.13, *p* = 0.896, nor was the direct effect, *B* = –0.08, (β = –0.06), *p* = 0.650, suggesting there was “no effect, non-mediation.” The indirect effect of the FIERS (<0.01) was about 4% of the total effect (0.075), and 0.0 times as large as the direct effect of SL-ASIA to BSQ (0.078). Thus, internalization completely accounted for the relation between acculturation and body shape dissatisfaction and eating disorder symptoms, respectively. Religiosity had neither direct nor indirect associations with any variable.

## Discussion

Relatively little is known about eating disorder risk in minoritized women in the United States (Rodgers et al., 2019; Burke et al., 2020), and even less has been established for women of SSEA descent. In this sample of SSEA women living in the United States, internalization completely accounted for the relationship of acculturation to both body image dissatisfaction and disordered eating, even when controlling for BMI and country of birth. By contrast, there were no significant relations between religiosity to the variables of interest. These results suggest that thin ideal internalization is a risk factor for body dissatisfaction and disordered eating for SSEA women, whereas acculturation and religiosity may not convey similar levels of risk. These findings are consistent with some past research on thin ideal internalization in SSEA women in high income countries (e.g., Mussap, 2009; Akoury et al., 2019; Goel et al., 2021), indicating that desiring the thin ideal increases the risk of body dissatisfaction and eating disorders regardless of ethnicity or culture of origin (Nouri et al., 2011; Doris et al., 2015; Warren and Akoury, 2020).

We hypothesized that greater acculturation would be linked directly and indirectly to greater body shape dissatisfaction and disordered eating symptoms. Though acculturation did not have *direct* associations with the dependent variables, it did have *indirect* associations. It appears as if greater acculturation corresponded to greater internalization of appearance ideals that, in turn, corresponded to greater eating disorder symptoms. These findings are in line with prior work showing no direct associations between acculturation and eating disorder risk in samples of SSEA women living in predominantly White cultures (Iyer and Haslam, 2003; Reddy and Crowther, 2007; Akoury et al., 2019). These results need to be replicated in a longitudinal design. In prior research, *difficulties* associated with adjusting to a new culture has been found to be a consistent risk factor for disordered eating (Iyer and Haslam, 2003; Reddy and Crowther, 2007; Akoury et al., 2019; Goel et al., 2021). Akoury et al. (2019) found a nuanced relationship with acculturation where *biculturalism* (maintaining an equal identity with one’s culture of origin and the dominant culture in which one lives) was associated with reduced eating disorder risk whereas *acculturative stress* (perceived difficulties one experiences transitioning to a new culture) predicted greater disordered eating. As in previous work, acculturation may have indirectly contributed to risk for disordered eating (i.e., by inflating internalization of appearance ideals), but was not a risk factor in-and-of itself.

Acculturation is not a homogenous experience. It may be ineffective to maintain acculturation as a linear variable where “more” acculturation predicts symptom quantity or severity, especially in the SSEA population. The SL-ASIA (Suinn et al., 1987), as used in the present study, only allows for measurement of acculturation in a linear manner. We chose this instrument for its wide use and tailored approach to acculturation for individuals of Asian descent. However, it is certainly possible that important aspects of acculturation were missed. A more nuanced measure may have captured important elements of acculturative stress that are directly related to eating disorder risk and/or the SSEA experience. For example, individual experiences of acculturation (e.g., acculturative stress, discrimination, bullying, and harassment) may also be important to examine in future research (Warren and Akoury, 2020).

It is unclear why internalization of appearance ideals entirely accounted for the variance in the present model. It may be that these appearance ideals are not as specific to predominantly White or affluent cultures as some suggest (Swami, 2015; Stojcic et al., 2020), and, instead, pressure for an ideal appearance (usually thinness) has been identified across industrialized, high income, and urbanized cultures (Yan and Bissell, 2014; Swami, 2015; Gorrell et al., 2019; Rodgers et al., 2020). Therefore, the appearance ideals of the dominant culture (i.e., the United States) may not be entirely different from a growing number of cultures of origin, including SSEA (Swami, 2015; Vaidyanathan et al., 2019; Karia et al., 2021). A related consideration in these findings is that we did not differentiate the type or origin of media consumed. Some work suggests that media consumption in SSEA countries, especially Bollywood films, is associated with similar patterns of body dissatisfaction as in White counterparts (Nagar and Virk, 2017). Importantly, the SATAQ-IG, as used in the present study, has been found to similarly index body comparisons to media consumption in SSEA media markets (Lewis-Smith et al., 2021). An advantage of the SATAQ-IG scale (specifically within the SATAQ-3) is that it does not denote which appearance ideal (e.g., weight, skin tone, hair color, etc.) one has internalized and, therefore, captures internalization more broadly than other instruments (Warren et al., 2013; Lewis-Smith et al., 2021). On the other hand, this broad measure does not allow for a closer analysis of media type consumed and whether, or how, it may be associated with internalization.^9^ It would be informative for future work to establish a more granular association of media type, origin of media, and eating disorder risk in diverse and/or minoritized individuals. As both culture and media change and evolve, it is important to maintain and update the instruments used to best measure the constructs of interest.

Another important consideration is the cultural variation of underlying motives for thinness. For White and East Asian women, a desire to be thin for aesthetic purposes is predominant whereas SSEA women tend to cite health motives (Lee et al., 2001; Rieger et al., 2001; Talukdar, 2012; Vaidyanathan et al., 2019; Ando et al., 2021). It is certainly possible that these motives persist even after relocating or immigrating (Warren and Akoury, 2020), or through intergenerational messaging if born in a predominantly White culture (Rhodes et al., 2016). These distinctions again highlight the importance of cultural sensitivity in eating disorder research, including underlying etiology of body image dissatisfaction.

Unlike internalization, religiosity had neither direct nor indirect relations with body dissatisfaction and disordered eating. This finding stands in contrast to some previous work that identified religiosity as a risk for disordered eating in samples of Hindu and Muslim women (Pengpid et al., 2015; Thomas et al., 2018; Goel et al., 2021). However, the samples in these studies were recruited from within SSEA countries; whereas all participants in the present sample were living in the United States. There may be a culturally specific aspect of religious adherence within those regions that does not translate to religious adherence for women who have relocated to the United States or who are first- or second- generation Americans. It may also be that our measurement of religiosity did not capture the components of religious observance that convey risk for disordered eating. The FIERS was validated in predominantly Christian (mainly Protestant) samples of respondents from the Southern United States (Feagin, 1964; Hood, 1971), with future validation that included more diverse, but still largely Judeo-Christian, respondents (Kirkpatrick, 1989; Genia, 1993). Similar to others (e.g., Hill and Dwiwardani, 2010; Fischer et al., 2016), we modified the instrument to be more inclusive, though these modifications may not have been sufficient to capture elements of religiosity relevant to the present heterogeneous, predominantly Hindu and Muslim sample. Past research has demonstrated that intrinsic religiosity—engaging in religious observance because it is personally meaningful and rewarding—is negatively associated with disordered eating and body dissatisfaction, whereas extrinsic religiosity—engaging in religious observance because for transactional or materialist purposes—is positively associated with disordered eating and body dissatisfaction (Akrawi et al., 2015). The FIERS was developed within a framework of capturing both intrinsic and extrinsic motives for religious adherence, and it may be these concepts and views of religion are culture-bound to American Protestants (Cohen and Hill, 2007; Flere and Lavrič, 2008). It was beyond the scope of this study to take a more granular approach to religion within the current sample (e.g., comparing religious identity between respondents). Future work should consider a more inclusive instrument and/or or one without intrinsic and extrinsic motives as a framework.

In contrast to previous work (e.g., Swami et al., 2014; Al-Mutawa et al., 2019), the Muslim women in the present sample who wore a Hijab or Niqab did not significantly differ on any measure from the Muslim women who did not. This finding should be interpreted with caution for several reasons. The sample size of Muslim women in this study was relatively small and this analysis was not central to our hypotheses. We did not collect data on modest dress more broadly, including covering of other body parts, that may have an influence of body image (Al-Mutawa et al., 2019). Importantly, our question about wearing a niqab or hijab was biased toward women who practice Islam; therefore, Hindu women who chose to wear a kameez or a veil to cover their chest or body would not have been captured. It is also worth noting that we were unable to identify any study that has assessed eating disorder risk as a function of modest dress in any religion besides Islam (e.g., Swami et al., 2014; Wilhelm et al., 2018) and Judaism (e.g., Geller et al., 2020). Finally, it may be possible that other variables interact with modest dress that can result in disordered eating risk, such as age, acculturation and acculturative stress, and marital status. It was beyond the scope of this study to examine modest dress further, but this is a potentially informative avenue for future research.

### Limitations

This study is one in a small body of research specifically probing psychosocial eating disorder risk in South and Southeast Asian women and the first to investigate thin ideal internalization and religiosity as putative mediators in that model. Nonetheless, there are some limitations that should be noted. We collapsed across South and Southeast Asian groups. Although the two groups did not differ meaningfully on any of the variables of interest and they share similar sociocultural histories and values (Allerton, 2009; Beteille, 2015; Evers, 2015), there are certainly differences between them that should be considered in future work. This study was cross-sectional, and participants were from the Mid-Atlantic United States, which limits generalizability and inferences about causality. We did not collect indices of socioeconomic status, which is an important variable to consider in future work. Though infrequently studied, previous work suggests that the relation of socioeconomic status, race, and eating disorder risk is complex and merits its own focused study (e.g., Mulders-Jones et al., 2017). Additionally, it was beyond the scope of this study to assess sub-scale-level analyses of these data (other than the SATAQ-IG). We used the sum score of the EAT-26, which has demonstrated validity and reliability in assessing eating disorder risk in non-clinical samples (e.g., Rogoza et al., 2016), though there is ongoing concern about this instrument and its factor structure (e.g., Papini et al., 2021). There also has been ongoing critique of the psychometrics and length of the BSQ (e.g., Pook et al., 2008). However, a preponderance of evidence suggests that the original 34-item measure provides reasonable psychometric indices when kept as a unitary scale, though verification of the cross-cultural validity of the instrument remains unconfirmed (Kling et al., 2019). Nevertheless, future work would do well to probe eating disorder risk with additional measures to provide more nuanced insights. We only inquired about modest dress for Muslims *via* the niqab, hijab, or burqa. Most available work assessing modest dress has included only Muslim (e.g., Swami et al., 2014; Wilhelm et al., 2018) or Jewish women (e.g., Geller et al., 2020), with a fairly consistent pattern of results: modest dress is associated with reduced eating disorder risk. However, this bias toward Islam and Judaism is a clear gap in the literature and more work is needed to determine if these findings are generalizable. Finally, we excluded men from analyses. Eating disorders in men has long been an understudied and poorly understood topic (Strother et al., 2012). Some work suggests that the intersectionality of race, acculturation and other psychosocial pressures can increase risk for disordered eating in SSEA men (Abbas et al., 2010; Warren and Akoury, 2020), which is an important avenue for future work.

It is worth emphasizing that cross-sectional data may induce bias in mediation analyses as this analysis assumes a linear and/or causal pathway (Maxwell et al., 2011; Shrout, 2011). We believe the tradeoff of reducing Type I error by addressing one atemporal model instead of numerous regression models outweighs the risk of this bias (Iacobucci et al., 2007; Zhao et al., 2010; Acock, 2013). We do not suggest temporal relations between these variables and do not make claims of causality: internalization may contribute to acculturation as much as the other way around. Although we can hypothesize temporal relationships, a prospective design would be necessary to establish this. Our goal in the present study was to establish putative relationships amongst these variables in a parsimonious way with minimal tests of association. Nonetheless, our results highlight the need to unpack “internalization of appearance ideals” and explore the role of religiosity in a more nuanced fashion. The relation of acculturation to eating behaviors can change over time (Alidu and Grunfeld, 2018), and it is important to evaluate these variables longitudinally.

## Conclusion

The contribution of acculturation to disordered eating is complex, and subtle cultural differences need to be handled with sensitivity. The present study contributes to the literature by focusing on risk for disordered eating attitudes and behaviors among SSEA women. The findings suggested that acculturation contributes to internalization of appearance ideal that may, in turn, contribute to risk of disordered eating. Acculturation may indirectly contribute to eating disorder risk by increasing internalization of appearance ideals, as promoted by the dominant White culture (Swami, 2015; Warren and Akoury, 2020). An important caveat to these findings is that they do not neatly reflect the results of the limited previous research and prospective work is needed to establish causal relationships. Much more research is needed to establish the unique experience of SSEA women that may contribute to disordered eating.

## Data Availability Statement

The original contributions presented in this study are included in the article/Supplementary Material. Data associated with this study is available at: https://osf.io/scg6j/.

## Ethics Statement

The studies involving human participants were reviewed and approved by Towson University; University of the Sciences. Written informed consent for participation was not required for this study in accordance with the national legislation and the institutional requirements.

## Author Contributions

SN: data curation, writing – original draft preparation, and writing – review and editing. EB: formal analysis, writing – original draft preparation, and writing – review and editing. MS: investigation, data curation, and writing – review and editing. AG: writing – review and editing. CT: conceptualization, methodology, investigation, and writing – review and editing. All authors contributed to the article and approved the submitted version.

## Conflict of Interest

The authors declare that the research was conducted in the absence of any commercial or financial relationships that could be construed as a potential conflict of interest.

## Publisher’s Note

All claims expressed in this article are solely those of the authors and do not necessarily represent those of their affiliated organizations, or those of the publisher, the editors and the reviewers. Any product that may be evaluated in this article, or claim that may be made by its manufacturer, is not guaranteed or endorsed by the publisher.

## Footnotes

1 According to the United Nations Statistical Division (UNSD): South Asia: Afghanistan, Bangladesh, Bhutan, India, Iran, Maldives, Nepal, Pakistan, and Sri Lanka; Southeastern Asia: Brunei, Cambodia, Indonesia, Laos, Malaysia, Myanmar, Philippines, Singapore, Thailand, and Vietnam (UNSD, 1999). Given the similarity in these two regions on the variables of interest, as well as underrepresentation of Southeastern Asia in this sample, we combined them here.

2 East Asia: China, Japan, Mongolia, North Korea, South Korea, and Taiwan (UNSD, 1999).

3 “Northern Asia” is not officially recognized by the UNSD. The Siberian region of Russia is the only geopolitical entity as a candidate for this designation.

4 Recruitment was not limited to SSEA individuals; university students completed a sub-group of the measures focused on body dissatisfaction and disordered eating for an unrelated study.

5 Data collection for this sample spanned 2009–2015, which predated the release of an updated (4th) version of the SATAQ (Schaefer et al., 2015). For the purposes of the present study, we also believe that the internalization of the appearance ideals is better measured by the SATAQ-3 which inquires specifically about a desire to appear like individuals in popular media, whereas the SATAQ-4 emphasizes a desire to be one, or a combination, of thinner, more muscular, and/or more attractive without mention of media or cultural pressure.

6 Best practices for bootstrapping with missing data for exogenous variables are to either remove the participants entirely or replace the data cautiously under certain conditions (Allison, 2003). Given the rarity of implausible values (*n* = 3), and that BMI was not a central component of the model, we opted to replace the data rather than omit the participants. Omitting these cases did not change significance or direction of the results (results not shown).

7 The target model is considered a reasonably good fit if: (a) the χ^2^ is not significant, (b) standardized root mean squared residual (SRMR) ⪅0.08; (c) root mean square error of approximation (RSMSEA) ⪅0.06; (d) comparative fit index (CFI) and (e) Tucker-Lewis Index (TLI) are both ⪆0.95.

8 “Western” comprised 70 respondents (63% of the sample): 68 born in the United States and two born in the United Kingdom; see Table 1.

9 As an exploratory measure, we conducted Spearman correlation analyses between the measures of interest in the current study and Factor 1 of the SL-ASIA, which indexes language familiarity and preference and includes items assessing type of media consumed (Suinn et al., 1992). We also included just the mean of items 10 (preferred music) and 11 (preferred movies). The SATAQ-IG significantly correlated to Factor 1 (ρ = 0.210, *p* = 0.027), and the BSQ at a trend-level (ρ = 0.185, *p* = 0.052). The association between the SATAQ-IG and the mean of questions 10 and 11 was not significant (ρ = 0.157, *p* = 0.10). Not surprisingly, those born in the United States or United Kingdom scored higher on this instrument than those born elsewhere, though the effect was small (ρ = 0.188 *p* = 0.047). No other correlation approached significance (| ρ| < 0.14, *p*s > 0.18). It may be that type or origin of media does not predict internalization and its sequelae so much as language ability and preference. Given the exploratory nature of this analysis, these findings should be interpreted with caution.

## Supplementary Material

Supplementary Table 1 Appendix A.

Supplementary Image 1 Appendix B.

## References

[B1] Abbas S. Damani S. Malik I. Button E. Aldridge S. Palmer R. (2010). A comparative study of South Asian and non-asian referrals to an eating disorders service in Leicester, UK. *Eur. Eat. Disord. Rev.* 18 404–409. 10.1002/erv.1033 20593482

[B2] Abraido-Lanza A. F. Armbrister A. N. Florez K. R. Aguirre A. N. (2006). Toward a theory-driven model of acculturation in public health research. *Am. J. Public Health* 96 1342–1346. 10.2105/AJPH.2005.064980 16809597 PMC1522104

[B3] Acock A. C. (2005). Working with missing values. *J. Marriage Fam.* 67 1012–1028. 10.1111/j.1741-3737.2005.00191.x

[B4] Acock A. C. (2013). *Discovering Structural Equation Modeling Using Stata.* College Station, TX: Stata Press Books.

[B5] Akgul S. Derman O. Kanbur N. O. (2014). Fasting during ramadan: a religious factor as a possible trigger or exacerbator for eating disorders in adolescents. *Int. J. Eat. Disord.* 47 905–910. 10.1002/eat.22255 24474707

[B6] Akoury L. M. Warren C. S. Culbert K. M. (2019). Disordered eating in Asian American women: sociocultural and culture-specific predictors. *Frontiers in Psychology* 10:1950. 10.3389/fpsyg.2019.01950 31551855 PMC6737071

[B7] Akrawi D. Bartrop R. Potter U. Touyz S. (2015). Religiosity, spirituality in relation to disordered eating and body image concerns: a systematic review. *J. Eat. Disord.* 3:29. 10.1186/s40337-015-0064-0 26279837 PMC4536728

[B8] Alidu L. Grunfeld E. (2018). A systematic review of acculturation, obesity and health behaviours among migrants to high-income countries. *Psychol. Health* 33 724–745. 10.1080/08870446.2017.1398327 29172700

[B9] Allerton C. (2009). Introduction: spiritual landscapes of Southeast Asia. *Anthropol. Forum* 19 235–251. 10.1080/00664670903278387

[B10] Allison P. D. (2003). Missing data techniques for structural equation modeling. *J. Abnorm. Psychol.* 112 545–557. 10.1037/0021-843X.112.4.545 14674868

[B11] Al-Mutawa N. Schuilenberg S. J. Justine R. Kulsoom Taher S. (2019). Modesty, objectification, and disordered eating patterns: a comparative study between veiled and unveiled muslim women residing in Kuwait. *Med. Principles Pract.* 28 41–47. 10.1159/000495567 30453295 PMC6558344

[B12] Ando K. Giorgianni F. E. Danthinne E. S. Rodgers R. F. (2021). Beauty ideals, social media, and body positivity: a qualitative investigation of influences on body image among young women in Japan. *Body Image* 38 358–369. 10.1016/j.bodyim.2021.05.001 34120098

[B13] Beteille A. (2015). “Asia, sociocultural overviews: South Asia,” in *International Encyclopedia of the Social & Behavioral Sciences*, 2nd Edn. ed. Wright J. D. (Amsterdam: Elsevier), 76–80. 10.1016/b978-0-08-097086-8.12019-7

[B14] Boyatzis C. J. Quinlan K. B. (2008). Women’s body image, disordered eating, and religion: a critical review of the literature. *Res. Soc. Sci. Study Relig.* 19 183–208. 10.1163/ej.9789004166462.i-299.61

[B15] Brown M. Cachelin F. M. Dohm F.-A. (2009). Eating disorders in ethnic minority women: a review of the emerging literature. *Curr. Psychiatry Rev.* 5 182–193. 10.2174/157340009788971119

[B16] Budiman A. Ruiz N. G. (2021). *Asian Americans are the Fastest-Growing Racial or Ethnic Group in the U.S. Pew Research Center.* Washington, DC: Pew Research Center.

[B17] Burke N. L. Schaefer L. M. Hazzard V. M. Rodgers R. F. (2020). Where identities converge: the importance of intersectionality in eating disorders research. *Int. J. Eat. Disord.* 53 1605–1609. 10.1002/eat.23371 32856342 PMC7722117

[B18] Cohen A. B. Hill P. C. (2007). Religion as culture: religious individualism and collectivism among American catholics, jews, and protestants. *J. Pers.* 75 709–742. 10.1111/j.1467-6494.2007.00454.x 17576356

[B19] Cooper P. J. Taylor M. J. Cooper Z. Fairbum C. G. (1987). The development and validation of the body shape questionnaire. *Int. J. Eat. Disord.* 6 485–494. 10.1016/j.eatbeh.2006.06.001 17336790

[B20] Cummins L. H. Simmons A. M. Zane N. W. (2005). Eating disorders in Asian populations: a critique of current approaches to the study of culture, ethnicity, and eating disorders. *Am. J. Orthopsychiatry* 75 553–574. 10.1037/0002-9432.75.4.553 16262514

[B21] Doris E. Shekriladze I. Javakhishvili N. Jones R. Treasure J. Tchanturia K. (2015). Is cultural change associated with eating disorders? A systematic review of the literature. *Eat. Weight Disord.* 20 149–160. 10.1007/s40519-015-0189-9 25894606

[B22] Downey R. G. King C. (1998). Missing data in likert ratings: a comparison of replacement methods. *J. Gen. Psychol.* 125 175–191. 10.1080/00221309809595542 9935342

[B23] Eekhout I. de Vet H. C. Twisk J. W. Brand J. P. de Boer M. R. Heymans M. W. (2014). Missing data in a multi-item instrument were best handled by multiple imputation at the item score level. *J. Clin. Epidemiol.* 67 335–342. 10.1016/j.jclinepi.2013.09.009 24291505

[B24] Evers H.-D. (2015). “Southeast asia: sociocultural aspects,” in *International Encyclopedia of the Social & Behavioral Sciences*, 2nd Edn. ed. Wright J. D. (Amsterdam: Elsevier), 70–74. 10.1016/b978-0-08-097086-8.12020-3

[B25] Feagin J. R. (1964). Prejudice and relegious types: a focused study of southern fundamentalists. *J. Sci. Study Relig.* 4 3–13. 10.2307/1385200

[B26] Fischer P. Greitemeyer T. Kastenmüller A. (2016). What do we think about muslims? The validity of westerners’ implicit theories about the associations between muslims’ religiosity, religious identity, aggression potential, and attitudes toward terrorism. *Group Process. Intergroup Relat.* 10 373–382. 10.1177/1368430207078697

[B27] Flere S. Lavrič M. (2008). Is intrinsic religious orientation a culturally specific American protestant concept? The fusion of intrinsic and extrinsic religious orientation among non-protestants. *Eur. J. Soc. Psychol.* 38 521–530. 10.1002/ejsp.437

[B28] Franzen L. Smith C. (2009). Acculturation and environmental change impacts dietary habits among adult Hmong. *Appetite* 52, 173–183. 10.1016/j.appet.2008.09.012 18848592

[B29] Garner D. M. Olmsted M. P. Bohr Y. Garfinkel P. E. (1982). The eating attitudes test: psychometric features and clinical correlates. *Psychol. Med.* 12 871–878. 10.1017/s0033291700049163 6961471

[B30] Geller S. Handelzalts J. Gelfat R. Arbel S. Sidi Y. Levy S. (2020). Exploring body image, strength of faith, and media exposure among three denominations of jewish women. *Curr. Psychol.* 39 1774–1784. 10.1007/s12144-018-9876-9

[B31] Genia V. (1993). A psychometric evaluation of the allport-ross i/e scales in a religiously heterogeneous sample. *J. Sci. Study Relig.* 32 284–290. 10.2307/1386667

[B32] Gerber L. Hill S. Manigault-Bryant L. (2015). Religion and fat = protestant christianity and weight loss? On the intersections of fat studies and religious studies. *Fat Stud.* 4 82–91. 10.1080/21604851.2015.1018071

[B33] Getz M. J. (2014). The myth of Chinese barbies: eating disorders in China including Hong Kong. *J. Psychiatr. Ment. Health Nurs.* 21 746–754. 10.1111/jpm.12115 25356465

[B34] Goel N. J. Thomas B. Boutté R. L. Kaur B. Mazzeo S. E. (2021). Body image and eating disorders among South Asian American women: what are we missing? *Qual. Health Res.* 31 2512–2521. 10.1177/10497323211036896 34382899

[B35] Gorrell S. Trainor C. Le Grange D. (2019). The impact of urbanization on risk for eating disorders. *Curr. Opin. Psychiatry* 32 242–247. 10.1097/YCO.0000000000000497 30724753 PMC6438744

[B36] Gulamhussein Q.-U.-A. Eaton N. R. (2015). Hijab, religiosity, and psychological wellbeing of muslim women in the United States. *J. Muslim Ment. Health* 9 25–40. 10.3998/jmmh.10381607.0009.202 26400043

[B37] Gutin I. (2018). In bmi we trust: reframing the body mass index as a measure of health. *Soc. Theory Health* 16 256–271. 10.1057/s41285-017-0055-0 31007613 PMC6469873

[B38] Hasan F. Latzer Y. Diedrichs P. C. Lewis-Smith H. (2021). A qualitative exploration of motivations for fasting and the impact of ramadan on eating behaviors and body image among young adult muslim women in the United Kingdom. *Eat. Behav.* 42:101545. 10.1016/j.eatbeh.2021.101545 34343839

[B39] Hasan S. (2012). “European colonization and the muslim majority countries: antecedents, approaches, and impacts,” in *The Muslim World in the 21st Century*, ed. Hasan S. (Dordrecht: Springer), 133–157. 10.1007/978-94-007-2633-8_7

[B40] Hayes-Bautista D. E. Bryant M. Yudell M. Hayes-Bautista T. M. Partlow K. Popejoy A. B. (2021). Office of management and budget racial/ethnic categories in mortality research: a framework for including the voices of racialized communities. *Am. J. Public Health* 111 S133–S140. 10.2105/AJPH.2021.306361 34314200 PMC8495649

[B41] Hill P. C. Dwiwardani C. (2010). “Measurement at the interface of psychiatry and religion: issues and existing measures,” in *Psychiatry and Religion: Beyond Boundaries*, eds Verhagen P. J. Van Praag H. M. Lopez-Ibor J. J. (New York, NY: Wiley), 319–339. 10.1002/9780470682203.ch18

[B42] Holland A. T. Palaniappan L. P. (2012). Problems with the collection and interpretation of Asian-American health data: omission, aggregation, and extrapolation. *Ann. Epidemiol.* 22 397–405. 10.1016/j.annepidem.2012.04.001 22625997 PMC4324759

[B43] Hood R. W. Jr. (1971). A comparison of the allport and feagin scoring procedures for intrinsic/extrinsic religious orientation. *J. Sci. Study Relig.* 10 370–374. 10.2307/1384783

[B44] Hu L. T. Bentler P. M. (1999). Cutoff criteria for fit indexes in covariance structure analysis: conventional criteria versus new alternatives. *Struct. Equ. Model.* 6 1–55. 10.1080/10705519909540118

[B45] Huang C.-C. (2019). “The influence of acculturation and weight-related behaviors on body mass index among Asian American ethnic subgroups,” in *Immigration and Health*, (Bingley: Emerald Publishing Limited).

[B46] Huisman M. (2000). Imputation of missing item responses: some simple techniques. *Qual. Quant.* 34 331–351. 10.1023/A:1004782230065

[B47] Huryk K. M. Drury C. R. Loeb K. L. (2021). Diseases of affluence? A systematic review of the literature on socioeconomic diversity in eating disorders. *Eat. Behav.* 43:101548. 10.1016/j.eatbeh.2021.101548 34425457

[B48] Iacobucci D. Saldanha N. Deng X. (2007). A meditation on mediation: evidence that structural equations models perform better than regressions. *J. Consum. Psychol.* 17 139–153. 10.1016/s1057-7408(07)70020-7

[B49] Iyer D. S. Haslam N. (2003). Body image and eating disturbance among South Asian-American women: the role of racial teasing. *Int. J. Eat. Disord.* 34 142–147. 10.1002/eat.10170 12772179

[B50] Karia S. Motwani S. Mandalia B. Desousa A. (2021). Eating disorders in India: an overview. *Ann. Indian Psychiatry* 5 12–17. 10.4103/aip.aip_27_21 33692645

[B51] Kirkpatrick L. A. (1989). A psychometric analysis of the allport-ross and feagin measures of intrinsic-extrinsic religious orientation. *Res. Soc. Sci. Study Relig.* 1 1–30.

[B52] Kling J. Kwakkenbos L. Diedrichs P. C. Rumsey N. Frisen A. Brandao M. P. (2019). Systematic review of body image measures. *Body Image* 30 170–211. 10.1016/j.bodyim.2019.06.006 31394462

[B53] Kodama K. Canetto S. S. (1995). Reliability and validity of the suinn-lew asian self-identity acculturation scale with Japanese temporary residents. *Psychologia* 38 17–21.

[B54] Komisarof A. Leong C. H. (2016). “Acculturation in East and Southeast Asia,” in *The Cambridge Handbook of Acculturation Psychology*, 2nd Edn, eds Sam D. L. Berry J. W. (Cambridge: Cambridge University Press), 248–271. 10.1017/cbo9781316219218.016

[B55] Kwon R. (2011). How the legacy of french colonization has shaped divergent levels of economic development in east Asia: a time-series cross-national analysis. *Sociol. Q.* 52 56–82. 10.1111/j.1533-8525.2010.01194.x

[B56] Ladd K. L. Spilka B. (2013). “Ritual and prayer: forms, functions, and relationships,” in *Handbook of the Psychology of Religion and Spirituality*, eds Paloutzian R. F. Park C. L. (New York, NY: The Guilford Press), 441–456.

[B57] Lee H. Y. Lock J. (2007). Anorexia nervosa in Asian-American adolescents: do they differ from their non-asian peers. *Int. J. Eat. Disord.* 40 227–231. 10.1002/eat.20364 17262816

[B58] Lee J. Ramakrishnan K. (2019). Who counts as Asian. *Ethnic Racial Stud.* 43 1733–1756. 10.1080/01419870.2019.1671600

[B59] Lee S. Lee A. M. Ngai E. Lee D. T. Wing Y. K. (2001). Rationales for food refusal in Chinese patients with Anorexia Nervosa. *Int. J. Eat. Disord.* 29 224–229. 10.1002/1098-108x(200103)29:2<224::aid-eat1012>3.0.co;2-r 11429985

[B60] Leong-Salobir C. (2011). *Food Culture in Colonial Asia: A Taste of Empire.* London: Routledge.

[B61] Lewis-Smith H. Garbett K. Chaudhry A. Uglik-Marucha N. Vitoratou S. Dhillon M. (2021). Adaptation and validation of the internalisation-general subscale of the sociocultural attitudes towards appearance questionnaire (sataq-3) in english among urban Indian adolescents. *Body Image* 36 254–262. 10.1016/j.bodyim.2020.12.004 33401203

[B62] Mack C. Su Z. Westrich D. (2018). “Types of missing data,” in *Managing Missing Data in Patient Registries: Addendum to Registries for Evaluating Patient Outcomes: A User’s Guide*, 3 Edn, (Rockville, MD: Agency for Healthcare Research and Quality (US)). Available online at: https://www.ncbi.nlm.nih.gov/books/NBK493614/29671990

[B63] Maxwell S. E. Cole D. A. Mitchell M. A. (2011). Bias in cross-sectional analyses of longitudinal mediation: partial and complete mediation under an autoregressive model. *Multivariate Behav. Res.* 46 816–841. 10.1080/00273171.2011.606716 26736047

[B64] Mehmetoglu M. (2018). Medsem: a stata package for statistical mediation analysis. *Int. J. Comput. Econ. Econometr.* 8 63–78. 10.1504/ijcee.2018.10007883 35009967

[B65] Monterubio G. E. Fitzsimmons-Craft E. E. Balantekin K. N. Sadeh-Sharvit S. Goel N. J. Laing O. (2020). Eating disorder symptomatology, clinical impairment, and comorbid psychopathology in racially and ethnically diverse college women with eating disorders. *Int. J. Eat. Disord.* 53 1868–1874. 10.1002/eat.23380 32918315 PMC7669650

[B66] Mulders-Jones B. Mitchison D. Girosi F. Hay P. (2017). Socioeconomic correlates of eating disorder symptoms in an Australian population-based sample. *PLoS One* 12:e0170603. 10.1371/journal.pone.0170603 28141807 PMC5283666

[B67] Mussap A. J. (2009). Acculturation, body image, and eating behaviours in muslim-Australian women. *Health Place* 15 532–539. 10.1016/j.healthplace.2008.08.008 18952486

[B68] Nagar I. Virk R. (2017). The struggle between the real and ideal: impact of acute media exposure on body image of young Indian women. *SAGE Open* 7:2158244017691327. 10.1177/2158244017691327

[B69] Nouri M. Hill L. G. Orrell-Valente J. K. (2011). Media exposure, internalization of the thin ideal, and body dissatisfaction: comparing Asian American and European American college females. *Body Image* 8 366–372. 10.1016/j.bodyim.2011.05.008 21775227

[B70] Office of Management and Budget (1997). *Revisions to the Standards for the Classification of Federal Data on Race and Ethnicity; 2 FR 58782, Oct. 30, 1997*. Federal Register. Available online at: https://www.govinfo.gov/content/pkg/FR-1997-10-30/pdf/97-28653.pdf

[B71] Panapasa S. Crabbe K. O. Kaholokula J. K. A. (2011). Efficacy of federal data: revised office of management and budget standard for native Hawaiian and other pacific islanders examined. *AAPI Nexus* 9 212–220. 10.17953/appc.9.1-2.cp21x04488016643 25360070 PMC4211287

[B72] Papini N. M. Jung M. Cook A. Lopez N. V. Ptomey L. T. Herrmann S. D. (2021). Psychometric properties of the 26-item eating attitudes test (EAT-26): an application of rasch analysis. *Preprint*10.1186/s40337-022-00580-3 PMC9069796 35509106

[B73] Pengpid S. Peltzer K. Ahsan G. U. (2015). Risk of eating disorders among university students in Bangladesh. *Int. J. Adolesc. Med. Health* 27 93–100. 10.1515/ijamh-2014-0013 25153370

[B74] Phillips C. E. King C. Kivisalu T. M. O’Toole S. K. (2016). A reliability generalization of the suinn-lew asian self-identity acculturation scale. *SAGE Open* 6 1–15. 10.1177/2158244016661748

[B75] Pook M. Tuschen-Caffier B. Brähler E. (2008). Evaluation and comparison of different versions of the body shape questionnaire. *Psychiatry Research* 158 67–73. 10.1016/j.psychres.2006.08.002 18037499

[B76] Preacher K. J. Coffman D. L. (2006). *Computing Power and Minimum Sample Size for Rmsea.* Available online at: http://www.quantpsy.org (accessed October 1, 2021).

[B77] Preacher K. J. Hayes A. F. (2004). Spss and sas procedures for estimating indirect effects in simple mediation models. *Behav. Res. Methods Instr. Comput.* 36 717–731. 10.3758/bf03206553 15641418

[B78] Preacher K. J. Hayes A. F. (2008). Asymptotic and resampling strategies for assessing and comparing indirect effects in multiple mediator models. *Behav. Res. Methods* 40 879–891. 10.3758/brm.40.3.879 18697684

[B79] Reddy S. D. Crowther J. H. (2007). Teasing, acculturation, and cultural conflict: psychosocial correlates of body image and eating attitudes among South Asian women. *Cult. Divers. Ethnic Minority Psychol.* 13 45–53. 10.1037/1099-9809.13.1.45 17227176

[B80] Rhodes K. Chan F. Prichard I. Coveney J. Ward P. Wilson C. (2016). Intergenerational transmission of dietary behaviours: a qualitative study of Anglo-Australian, Chinese-Australian and Italian-Australian three-generation families. *Appetite* 103 309–317. 10.1016/j.appet.2016.04.036 27133550

[B81] Richards P. S. Weinberger-Litman S. L. Susov S. Berrett M. E. (2013). “Religiousness and spirituality in the etiology and treatment of eating disorders,” in *APA Handbook of Psychology, Religion, and Spirituality (Vol 2): An Applied Psychology of Religion and Spirituality*, Vol. 2 eds Pargament K. I. Mahoney A. Shafranske E. P. (Washington, DC: American Psychological Association), 319–333. 10.1037/14046-016

[B82] Rieger E. Touyz S. W. Swain T. Beumont P. J. (2001). Cross-cultural research on Anorexia Nervosa: assumptions regarding the role of body weight. *Int. J. Eat. Disord.* 29 205–215. 10.1002/1098-108x(200103)29:2<205::aid-eat1010>3.0.co;2-1 11429983

[B83] Rodgers R. F. Berry R. Franko D. L. (2018). Eating disorders in ethnic minorities: an update. *Curr. Psychiatry Rep.* 20:90. 10.1007/s11920-018-0938-3 30155577

[B84] Rodgers R. F. Donovan E. Cousineau T. M. McGowan K. Yates K. Cook E. (2019). Ethnic and racial diversity in eating disorder prevention trials. *Eat. Disord.* 27 168–182. 10.1080/10640266.2019.1591824 31084423

[B85] Rodgers R. F. Fuller-Tyszkiewicz M. Markey C. Granero-Gallegos A. Sicilia A. Caltabiano M. (2020). Psychometric properties of measures of sociocultural influence and internalization of appearance ideals across eight countries. *Body Image* 35 300–315. 10.1016/j.bodyim.2020.09.016 33181386

[B86] Rodgers R. F. Peterson K. E. Hunt A. T. Spadano-Gasbarro J. L. Richmond T. K. Greaney M. L. (2017). Racial/ethnic and weight status disparities in dieting and disordered weight control behaviors among early adolescents. *Eat. Behav.* 26 104–107. 10.1016/j.eatbeh.2017.02.005 28226307

[B87] Rogoza R. Brytek-Matera A. Garner D. (2016). Analysis of the eat-26 in a non-clinical sample. *Arch. Psychiatry Psychother.* 18 54–58. 10.12740/app/63647

[B88] Schaefer L. M. Burke N. L. Thompson J. K. Dedrick R. F. Heinberg L. J. Calogero R. M. (2015). Development and validation of the sociocultural attitudes towards appearance questionnaire-4 (sataq-4). *Psychol. Assess.* 27 54–67. 10.1037/a0037917 25285718

[B89] Shrout P. E. (2011). Commentary: mediation analysis, causal process, and cross-sectional data. *Multiv. Behav. Res.* 46 852–860. 10.1080/00273171.2011.606718 26736049

[B90] Shrout P. E. Bolger N. (2002). Mediation in experimental and nonexperimental studies: new procedures and recommendations. *Psychol. Methods* 7 422–445. 10.1037//1082-989x.7.4.422 12530702

[B91] Siebenhütter S. (2019). “Sociocultural influences on linguistic geography: religion and language in southeast asia,” in *Handbook of the Changing World Language Map*, eds Brunn S. Kehrein R. (Cham: Springer), 1–19. 10.1007/978-3-319-73400-2_84-1

[B92] Smolak L. (2006). “Body image,” in *Handbook of Girls’ and Women’s Psychological Health: Gender and Well-Being Across the Lifespan*, eds Worell J. Goodheart C. D. (Oxford: Oxford University Press), 69–76.

[B93] Spangler D. L. (2010). Heavenly bodies: religious issues in cognitive behavioral treatment of eating disorders. *Cogn. Behav. Pract.* 17 358–370. 10.1016/j.cbpra.2009.05.004

[B94] Stojcic I. Dong X. Ren X. (2020). Body image and sociocultural predictors of body image dissatisfaction in croatian and Chinese women. *Front. Psychol.* 11:731. 10.3389/fpsyg.2020.00731 32435214 PMC7218091

[B95] Strother E. Lemberg R. Stanford S. C. Turberville D. (2012). Eating disorders in men: underdiagnosed, undertreated, and misunderstood. *Eat. Disord.* 20 346–355. 10.1080/10640266.2012.715512 22985232 PMC3479631

[B96] Suinn R. M. Ahuna C. Khoo G. (1992). The suinn-lew asian self-identity acculturation scale: concurrent and factorial validation. *Educ. Psychol. Meas.* 52 1041–1046. 10.1177/0013164492052004028

[B97] Suinn R. M. Rickard-Figueroa K. Lew S. Vigil P. (1987). The Suinn-lew Asian self-identity acculturation scale: an initial report. *Educ. Psychol. Meas.* 47 401–407. 10.1177/0013164487472012

[B98] Sussman N. M. Truong N. Lim J. (2007). Who experiences “America the beautiful”?: ethnicity moderating the effect of acculturation on body image and risks for eating disorders among immigrant women. *Int. J. Intercult. Relat.* 31 29–49. 10.1016/j.ijintrel.2006.03.003

[B99] Swami V. (2015). Cultural influences on body size ideals. *Eur. Psychol.* 20 44–51. 10.1027/1016-9040/a000150

[B100] Swami V. (2016). Change in risk factors for eating disorder symptomatology in malay students Sojourning in the United Kingdom. *Int. J. Eat. Disord.* 49 695–700. 10.1002/eat.22509 26876737

[B101] Swami V. Miah J. Noorani N. Taylor D. (2014). Is the hijab protective? An investigation of body image and related constructs among British muslim women. *Br. J. Psychol.* 105 352–363. 10.1111/bjop.12045 25040005

[B102] Talukdar J. (2012). Thin but not skinny: women negotiating the “never too thin” body ideal in urban India. *Womens Stud. Int. Forum* 35 109–118. 10.1016/j.wsif.2012.03.002

[B103] Tareen A. Hodes M. Rangel L. (2005). Non-fat-phobic anorexia nervosa in British south Asian adolescents. *Int. J. Eat. Disord.* 37 161–165. 10.1002/eat.20080 15732077

[B104] Thomas J. O’Hara L. Tahboub-Schulte S. Grey I. Chowdhury N. (2018). Holy anorexia: eating disorders symptomatology and religiosity among muslim women in the United Arab Emirates. *Psychiatry Res.* 260 495–499. 10.1016/j.psychres.2017.11.082 29291574

[B105] Thompson J. K. van den Berg P. Roehrig M. Guarda A. S. Heinberg L. J. (2004). The sociocultural attitudes towards appearance scale-3 (sataq-3): development and validation. *Int. J. Eat. Disord.* 35 293–304. 10.1002/eat.10257 15048945

[B106] UNSD (1999). *Standard Country or Area Codes for Statistical Use, 1999 (Revision 4), Document m49.* New York, NY: UNSD.

[B107] Vaidyanathan S. Kuppili P. P. Menon V. (2019). Eating disorders: an overview of Indian research. *Indian J. Psychol. Med.* 41 311–317. 10.4103/IJPSYM.IJPSYM_461_18 31391662 PMC6657488

[B108] Walker H. D. (2012). *East Asia: A new History.* Bloomington, IN: AuthorHouse.

[B109] Warren C. S. (2012). Body area dissatisfaction in white, black and Latina female college students in the USA: an examination of racially salient appearance areas and ethnic identity. *Ethnic Racial Stud.* 37 537–556. 10.1080/01419870.2012.716520

[B110] Warren C. S. Akoury L. M. (2020). Emphasizing the “cultural” in sociocultural: a systematic review of research on thin-ideal internalization, acculturation, and eating pathology in us ethnic minorities. *Psychol. Res. Behav. Manag.* 13 319–330. 10.2147/PRBM.S204274 32280289 PMC7132000

[B111] Warren C. S. Gleaves D. H. Rakhkovskaya L. M. (2013). Score reliability and factor similarity of the sociocultural attitudes towards appearance questionnaire-3 (sataq-3) among four ethnic groups. *J. Eat. Disord.* 1 1–8. 10.1186/2050-2974-1-14 24999395 PMC4081787

[B112] Weir C. B. Jan A. (2021). *BMI Classification Percentile and cut off Points.* St. Petersburg, FL: StatPearls Publishing. 31082114

[B113] Wilhelm L. Hartmann A. S. Becker J. C. Kisi M. Waldorf M. Vocks S. (2018). Body covering and body image: a comparison of veiled and unveiled muslim women, christian women, and atheist women regarding body checking, body dissatisfaction, and eating disorder symptoms. *J. Relig. Health* 57 1808–1828. 10.1007/s10943-018-0585-3 29468534

[B114] Winer E. S. Cervone D. Bryant J. McKinney C. Liu R. T. Nadorff M. R. (2016). Distinguishing mediational models and analyses in clinical psychology: atemporal associations do not imply causation. *J. Clin. Psychol*. 72, 947–955. 10.1002/jclp.22298 27038095

[B115] Yan Y. Bissell K. (2014). The globalization of beauty: how is ideal beauty influenced by globally published fashion and beauty magazines? *J. Intercult. Commun. Res.* 43 194–214. 10.1080/17475759.2014.917432

[B116] Zhao X. Lynch J. G. Chen Q. (2010). Reconsidering baron and kenny: myths and truths about mediation analysis. *J. Consum. Res.* 37 197–206. 10.1086/651257

