# Supplementary material for: Internalization of Appearance Ideals and Not Religiosity Indirectly Impacts the Relationship Between Acculturation and Disordered Eating Risk in South and Southeast Asian Women Living in the United States
Source: Front Psychol. 2022 Jul 18;13:843717. doi: 10.3389/fpsyg.2022.843717 (PMC9341433; doi:10.3389/fpsyg.2022.843717)
Supplement: Supplementary Table 1 — Appendix A. [file Table_1.docx]

| Appendix A. Summary statistics for direct and indirect effects in the path analysis. | | | | | | | | |  |
| --- | --- | --- | --- | --- | --- | --- | --- | --- | --- |
|  | | B | SE of B | β | *z* | 95% CI Lower | 95% CI Upper | | |
| Direct Effects | | | | | | | | |  |
| BMI🡪 | |  |  |  |  |  | |  | |
|  | SATAQ-IG | -0.17 | 0.18 | -0.09 | -0.92 | -0.52 | 0.19 | | |
|  | FIERS | -0.20 | 0.25 | -0.07 | -0.77 | -0.70 | 0.30 | | |
|  | BSQ | 3.38*** | 0.94 | 0.34 | 3.61 | 0.99 | 4.47 | | |
|  | EAT | 0.27 | 0.27 | 0.10 | 1.03 | -0.34 | 0.66 | | |
|  | | | | | | | | |  |
| Born West. 🡪 | |  |  |  |  |  |  | | |
|  | SATAQ-IG | 2.14 | 1.67 | 0.13 | 1.28 | -1.13 | 5.42 | | |
|  | FIERS | 0.18 | 2.86 | 0.01 | 0.06 | -5.44 | 5.79 | | |
|  | BSQ | -22.32*** | 6.20 | -0.25 | -3.60 | -32.00 | 1.61 | | |
|  | EAT | -1.90 | 2.05 | -0.08 | -0.93 | -4.78 | 3.79 | | |
|  |  |  |  |  |  |  |  | | |
| *Notes*: 95% CI and statistical significance were calculated with bias corrected bootstraps using 1000 replications on the unstandardized values; B = unstandardized coefficient; β = standardized coefficient; BMI = Body Mass Index; Born West. = Born in either the United States (*n* = 68) or United Kingdom (*n* = 2) compared to born elsewhere (*n* = 42) dummy (*n* = 2) dummy coded as 1 = Yes, 2 = No, (negative coefficients indicate greater scores for “Western”-born individuals); SL-ASIA = Suinn-Lew Asian Self Identity Acculturation; SATAQ-IG = Sociocultural Attitudes Toward Appearance Questionnaire-Internalization General subscale; FIERS = Feagin Intrinsic-Extrinsic Religiosity Scale; BSQ = Body Shape Questionnaire; EAT = Eating Attitudes Test; note that it was beyond the scope of this study to analyze the indirect effects of BMI and place of birth to endogenous variables, but those analyses can be made available upon request;  * *p* < .05, ** *p* < .01, *** *p* < .001 | | | | | | | | |  |
