# Supplementary material for: Internalization of Appearance Ideals and Not Religiosity Indirectly Impacts the Relationship Between Acculturation and Disordered Eating Risk in South and Southeast Asian Women Living in the United States
Source: Front Psychol. 2022 Jul 18;13:843717. doi: 10.3389/fpsyg.2022.843717 (PMC9341433; doi:10.3389/fpsyg.2022.843717)
Supplement: Supplementary Table 2 — Appendix C. [file Table_2.docx]

| Appendix C: Comparisons of the variables of interest with Muslim women from the current sample who reported wearing a hijab or niqab vs those who do not | | | | | | | | |
| --- | --- | --- | --- | --- | --- | --- | --- | --- |
|  | *n* | | M (SD)/*n* (%) | |  |  |  |  |
|  | No Wear | Wear | No Wear | Wear | *t*/*χ*^2^ | df | *p* | *d/v* |
| SL-ASIA | 25 | 20 | 59.32 (6.57) | 62.05 (11.22) | -1.02^a^ | 29.12^a^ | .343 | -.30 |
| SATAQ-IG | 25 | 20 | 91.36 (22.27) | 85.05 (25.21) | 0.89 | 43 | .378 | .27 |
| FIERS | 21^b^ | 16^b^ | 55.14 (13.71) | 52.00 (10.63) | 0.76 | 35 | .453 | .25 |
| BSQ | 25 | 20 | 82.36 (43.38) | 82.75 (40.43) | -0.03 | 43 | .976 | -.01 |
| EAT | 25 | 20 | 10.44 (10.86) | 12.05 (11.96) | -0.47 | 43 | .639 | -.14 |
| BMI | 25 | 20 | 25.26 (3.56) | 23.85 (4.94) | -0.89 | 43 | .377 | -.27 |
| Age | 25 | 20 | 25.12 (6.44) | 28.3 (8.09) | -1.47 | 43 | .149 | -.44 |
| Born West. | 25 | 20 | 14 (56%) | 6 (30%) | 3.04 | 1 | .081 | .26 |
| *Notes:* SL-ASIA = Suinn-Lew Asian Self Identity Acculturation; SATAQ-IG = Sociocultural Attitudes Toward Appearance Questionnaire-Internalization General subscale; FIERS = Feagin Intrinsic-Extrinsic Religiosity Scale; BSQ = Body Shape Questionnaire; EAT = Eating Attitudes Test; BMI = Body Mass Index; Born West. = proportion of respondents born in the US (*n*  = 24) or UK (*n* = 1); df = degrees of freedom  ᵃ Levene's test was significant (*p* < .05), suggesting a violation of the assumption of equal variances, and Welch’s *t*-test is presented here instead;  ^b^ Four wearers and non-wearers (each) of hijab/niqab did not complete the FIERS | | | | | | | | |
