# Supplementary material for: Internalization of Appearance Ideals and Not Religiosity Indirectly Impacts the Relationship Between Acculturation and Disordered Eating Risk in South and Southeast Asian Women Living in the United States
Source: Front Psychol. 2022 Jul 18;13:843717. doi: 10.3389/fpsyg.2022.843717 (PMC9341433; doi:10.3389/fpsyg.2022.843717)
Supplement: Supplementary Table 3 — Appendix D. [file Table_3.docx]

| Appendix D.1. Results of One-Way ANOVA and *χ*^2^ comparing four religious groups (Christian, Muslim, Hindu, and “Other”) on the variables of interest in the current study | | | | | |
| --- | --- | --- | --- | --- | --- |
|  | *F*/*χ*^2^ | df_1_ | df_2_ | *p* | *ω*^2^/*V* |
| SL-ASIA | 1.91 | 3 | 108 | .139 | .047 |
| SATAQ-IG | 0.18 | 3 | 107 | .907 | .048 |
| FIERS^a^ | 3.02 | 3 | 95 | .039 | .111 |
| BSQ | 1.64 | 3 | 107 | .193 | .035 |
| EAT | 0.49 | 3 | 49.0 | .694 | .030 |
| Age | 9.86 | 3 | 108 | < .001 | .316 |
| BMI^b^ | 0.15 | 3 | 105 | .929 | .052 |
| Born West | 13.0 | 3 | -- | .005 | .340 |
| *Notes:* SL-ASIA = Suinn-Lew Asian Self Identity Acculturation; SATAQ-IG = Sociocultural Attitudes Toward Appearance Questionnaire-Internalization General subscale; FIERS = Feagin Intrinsic-Extrinsic Religiosity Scale; BSQ = Body Shape Questionnaire; EAT = Eating Attitudes Test; BMI = Body Mass Index; Born West. = proportion of respondents born in the US (*n*  = 24) or UK (*n* = 1); df = degrees of freedom  ^a^ Excludes 13 participants who did not complete the FIERS  ^b^ Excludes the three individuals who did not provide plausible values for a BMI | | | | | |

| Appendix D.2. Descriptive statistics of the four religious groups on the variables of interest. | | | |
| --- | --- | --- | --- |
|  | Religion | N | M (SD) |
| SL-ASIA | Christian | 20 | 62.7 (7.12) |
|  | Hindu | 28 | 61.8 (8.63) |
|  | Muslim | 45 | 60.5 (8.93) |
|  | Other | 19 | 65.8 (7.61) |
| SATAQ-IG | Christian | 20 | 28.8 (7.71) |
|  | Hindu | 27 | 27.2 (8.39) |
|  | Muslim | 45 | 28.0 (8.4) |
|  | Other | 19 | 28.7 (8.07) |
| FIERS ^a^ | Christian | 19 | 56.1 (12.47) |
|  | Hindu | 25 | 59 (9.46) |
|  | Muslim | 37 | 53.8 (12.41) |
|  | Other | 18 | 65.3 (14.48) |
| BSQ | Christian | 20 | 95.3 (45.95) |
|  | Hindu | 27 | 97.6 (40.77) |
|  | Muslim | 45 | 82.5 (41.62) |
|  | Other | 19 | 105.4 (40.06) |
| EAT | Christian | 20 | 10.7 (10.15) |
|  | Hindu | 28 | 13.9 (13.35) |
|  | Muslim | 45 | 11.2 (11.26) |
|  | Other | 19 | 13.6 (11.59) |
| Age | Christian | 20 | 19.9 (2.46) |
|  | Hindu | 28 | 21.5 (4.84) |
|  | Muslim | 45 | 26.5 (7.31) |
|  | Other | 19 | 20.7 (4.48) |
| BMI ^b^ | Christian | 20 | 22.5 (3.57) |
|  | Hindu | 27 | 23.4 (5.68) |
|  | Muslim | 44 | 23.0 (4.27) |
|  | Other | 18 | 23.0 (4.17) |
| Born West.^c^ | Christian | 20 | 14 (70%) |
|  | Hindu | 28 | 19 (68% |
|  | Muslim | 45 | 20 (44%) |
|  | Other | 19 | 17 (90a%) |
| *Notes:* SL-ASIA = Suinn-Lew Asian Self Identity Acculturation; SATAQ-IG = Sociocultural Attitudes Toward Appearance Questionnaire-Internalization General subscale; FIERS = Feagin Intrinsic-Extrinsic Religiosity Scale; BSQ = Body Shape Questionnaire; EAT = Eating Attitudes Test; BMI = Body Mass Index; Born West. = proportion of respondents born in the US (*n*  = 24) or UK (*n* = 1); df = degrees of freedom  ^a^ Excludes 13 participants who did not complete the FIERS ^b^ Excludes the three individuals who did not provide plausible values for a BMI ^c^ Values are presented as *n* (%) and reflect the percentage of the religious group born in the United States or United Kingdom. | | | |

Appendix D.3: narrative of the results of the four religious groups in the current study

As shown above, there were three significant differences as a function of religion. First, there was a main effect of religion on the FIERS. Post-hoc comparisons showed the the “Other” group scored significantly lower than the Muslim group on this assessment, *M*_diff_ = 11.49, *t*(95) = 2.89, *p* = .035. This difference was likely driven by the four identified atheists: removal of this group resulted in a null main effect, *F* (3,92) = 1.98, *p* = .138, *ω*^2^ = .061.

Next, there was a main effect of age, wherein the Muslim group was significantly older than the Christian group, *M*_diff_ = 6.68, *t* (108) = 4.39, *p* < .001, the Hindu group, *M*_diff_ = 5.03, *t* (108) = 3.69, *p* = .002, and the “Other” group, *M*_diff_ = 5.80, *t* (108) = 3.74, *p* = .002.

Finally, there was a main effect birthplace (Born in the US or UK vs. elsewhere), whereby Muslim respondents were overrepresented in the “born elsewhere” category, and the “Other” group was overrepresented in the group that was born in the US or UK.
